# Impact of sex and gender on post-COVID-19 syndrome, Switzerland, 2020 

**DOI:** 10.2807/1560-7917.ES.2024.29.2.2300200

**Published:** 2024-01-11

**Authors:** Caroline E Gebhard, Claudia Sütsch, Pimrapat Gebert, Bianca Gysi, Susan Bengs, Atanas Todorov, Manja Deforth, Philipp K Buehler, Alexander Meisel, Reto A Schuepbach, Annelies S Zinkernagel, Silvio D Brugger, Claudio Acevedo, Dimitri Patriki, Benedikt Wiggli, Jürg H Beer, Andrée Friedl, Raphael Twerenbold, Gabriela M Kuster, Hans Pargger, Sarah Tschudin-Sutter, Joerg C Schefold, Thibaud Spinetti, Chiara Henze, Mina Pasqualini, Dominik F Sager, Lilian Mayrhofer, Mirjam Grieder, Janna Tontsch, Fabian C Franzeck, Pedro D Wendel Garcia, Daniel A Hofmaenner, Thomas Scheier, Jan Bartussek, Achi Haider, Muriel Grämer, Nidaa Mikail, Alexia Rossi, Núria Zellweger, Petra Opić, Angela Portmann, Roland von Känel, Aju P Pazhenkottil, Michael Messerli, Ronny R Buechel, Philipp A Kaufmann, Valerie Treyer, Martin Siegemund, Ulrike Held, Vera Regitz-Zagrosek, Catherine Gebhard

**Affiliations:** 1 Intensive Care Unit, University Hospital Basel, University of Basel, Basel, Switzerland; 2 Department of Nuclear Medicine, University Hospital Zurich, University of Zurich, Zurich, Switzerland; 3 Center for Molecular Cardiology, University of Zurich, Schlieren, Switzerland; 4 Institute of Biometry and Clinical Epidemiology, Charité - Universitätsmedizin Berlin, Berlin, Germany; 5 Department of Biostatistics at Epidemiology, Biostatistics and Prevention Institute, University of Zurich, Zurich, Switzerland; 6 Institute of Intensive Care, University Hospital Zurich, University of Zurich, Zurich, Switzerland; 7 Department of Infectious Diseases and Hospital Epidemiology, University Hospital Zurich, University of Zurich, Zurich, Switzerland; 8 Department of Cardiology, University Hospital Zurich, Zurich, Switzerland; 9 Department of Internal Medicine, Cantonal Hospital of Baden, Baden, Switzerland; 10 Department of Cardiology, University Hospital Basel, Basel, Switzerland; 11 Department of Cardiology and University Center of Cardiovascular Science, University Heart and Vascular Center Hamburg, Hamburg, Germany; 12 Department of Biomedicine, University of Basel, Basel, Switzerland; 13 Division of Infectious Diseases and Hospital Epidemiology, University of Basel, Basel, Switzerland; 14 Department of Intensive Care Medicine, Inselspital Bern University Hospital, University of Bern, Bern, Switzerland; 15 Department of Informatics, University Hospital Basel, Basel, Switzerland; 16 Department of Quantitative Biomedicine, University of Zurich, Zurich, Switzerland; 17 Division of Nuclear Medicine and Molecular Imaging, Massachusetts General Hospital, and Department of Radiology, Harvard Medical School, Boston, Massachusetts, United States; 18 Department of Consultation-Liaison Psychiatry and Psychosomatic Medicine, University Hospital Zurich, University of Zurich, Zurich, Switzerland; 19 Institute of Gender in Medicine (GiM), Charité - Universitätsmedizin Berlin, Berlin, Germany; * These authors contributed equally

**Keywords:** SARS-CoV-2, Gender, Sex, Women, Post-COVID-19, Long-COVID, Long-Haulers

## Abstract

**Background:**

Women are overrepresented among individuals with post-acute sequelae of SARS-CoV-2 infection (PASC). Biological (sex) as well as sociocultural (gender) differences between women and men might account for this imbalance, yet their impact on PASC is unknown.

**Aim:**

We assessed the impact of sex and gender on PASC in a Swiss population.

**Method:**

Our multicentre prospective cohort study included 2,856 (46% women, mean age 44.2 ± 16.8 years) outpatients and hospitalised patients with PCR-confirmed SARS-CoV-2 infection.

**Results:**

Among those who remained outpatients during their first infection, women reported persisting symptoms more often than men (40.5% vs 25.5% of men; p < 0.001). This sex difference was absent in hospitalised patients. In a crude analysis, both female biological sex (RR = 1.59; 95% CI: 1.41–1.79; p < 0.001) and a score summarising gendered sociocultural variables (RR = 1.05; 95% CI: 1.03–1.07; p < 0.001) were significantly associated with PASC. Following multivariable adjustment, biological female sex (RR = 0.96; 95% CI: 0.74–1.25; p = 0.763) was outperformed by feminine gender-related factors such as a higher stress level (RR = 1.04; 95% CI: 1.01–1.06; p = 0.003), lower education (RR = 1.16; 95% CI: 1.03–1.30; p = 0.011), being female and living alone (RR = 1.91; 95% CI: 1.29–2.83; p = 0.001) or being male and earning the highest income in the household (RR = 0.76; 95% CI: 0.60–0.97; p = 0.030).

**Conclusion:**

Specific sociocultural parameters that differ in prevalence between women and men, or imply a unique risk for women, are predictors of PASC and may explain, at least in part, the higher incidence of PASC in women. Once patients are hospitalised during acute infection, sex differences in PASC are no longer evident.

**Key public health message**

**What did you want to address in this study?**

Women are more often affected by post-COVID symptoms than men. We sought to assess whether biological (sex) or sociocultural (gender) differences between women and men account for this imbalance.

**What have we learnt from this study?**

Sociocultural parameters that differ between women and men are risk predictors of post-COVID symptoms and may explain the female propensity towards a higher risk of those.

**What are the implications of your findings for public health?**

Currently, international guidelines suggest an approach to treat post-COVID effects based on symptoms, however, our data imply that a tailored gender-sensitive approach of healthcare services may be required to support the needs of affected individuals.

## Introduction

Severe acute respiratory syndrome coronavirus 2 (SARS-CoV-2) infection can cause a prolonged disease course beyond acute COVID-19 [1]. The clinical presentation of these post-acute sequelae of SARS-CoV-2 infection (PASC) includes a variety of fluctuating and unpredictable somatic symptoms persisting even beyond 12 months after initial infection, thereby creating a rising healthcare and economic burden [2-4]. In fact, recent data from the United States (US) indicate a considerable impact of PASC on the labour market, with 2–4 million individuals (out of 16 million working-age Americans affected by PASC) being on sick leave [5], while data from the UK showed an increase of 0.5 million people being out of the labour market because of long-term sickness from 2019 to 2022 [6]. Worldwide, at least 65 million individuals are estimated to have PASC, with a daily increase in cases [7]. Despite substantial efforts to identify pathophysiological mechanisms and risk factors of PASC, current diagnostic and treatment options are insufficient in dealing with this condition.

Although mortality and morbidity, such as intensive care unit admission, from acute COVID-19 infection is substantially lower in women than men, women are overrepresented among patients with PASC [8-13]. Accordingly, factors increasing the risk of severe acute COVID-19, such as advanced age or male sex, do not also increase the risk of PASC [14]. The causes for the differential sex and gender distribution in acute vs chronic COVID-19 remain enigmatic. Factors beyond innate sex, such as sociocultural gender, have been widely ignored in analysing the causes of sex and gender disparities in COVID-19 outcomes [15], an omission that has been criticised by several institutions including the Canadian Institutes of Health Research and the European Parliament [16,17].

We sought to assess the impact of social context, gender and behaviours in addition to biological data on PASC in a large and well-characterised multi-centre cohort in Switzerland comprising both hospitalised patients and outpatients with confirmed SARS-CoV-2 infection.

## Methods

### Study design and procedures

Our study is based on data from patients of the Swiss COGEN cohort study, a prospective, observational cohort of individuals who were diagnosed with PCR-confirmed SARS-CoV-2 infection between February and December 2020 at one of four Swiss study sites. Eligible patients were adults aged ≥ 18 years at follow-up who survived acute COVID-19 infection, residing in Switzerland during primary SARS-CoV-2 infection, fluent in German, English, French or Italian and able to provide written informed consent. After a minimum follow-up time of 12 weeks (based on current definitions of PASC [1]), each participant was contacted by telephone and asked to complete a questionnaire either by phone, email or on paper. Of 5,938 patients, 3,005 individuals (patients directly (n = 2,996) or their legal representatives (n = 9)) completed the questionnaire after giving informed consent; we append a flowchart of patient selection in Supplementary Figure S1. We obtained clinical data and laboratory data from electronic medical records containing information about demographic characteristics (age, sex), cardiovascular risk factors (including diabetes mellitus, hypertension, dyslipidaemia, family history of coronary artery disease, smoking, and obesity), symptoms and date of symptom onset, medication, pre-existing comorbidities, data on weight and height, and disease severity of COVID-19 classified according to symptoms and necessity of in-hospital (normal ward, intermediate or intensive care) treatment. Data on vital signs, respiratory parameters and organ support measures were gathered within the first 24 h of and during hospitalisation (worst value/highest level of organ support). We analysed stored blood samples available from patients who consented to provide blood samples for biobank storage for circulating hormone levels (testosterone, oestradiol, progesterone and cortisol). These blood samples were drawn from hospitalised patients at the first day of hospitalisation.

### Assessment of gender

Gender consists of four interrelated dimensions (definition provided by the World Health Organization [18] and the Women Health Research Network of the Canadian Institute of Health Research [19]) encompassing gender roles (e.g. child care), gender identity (a personal conception of oneself as man or woman), ‘gender relationships’ (e.g. social support), and ‘institutionalised gender’ (e.g. education level, personal income). Currently, there is no academic consensus on how to define the construct ‘gender’ as different approaches to operationalise gender have been proposed [20]. Consequently, there is no gold standard for a measure of gender. However, Pelletier et al. have previously introduced and validated a methodological approach where a composite gender score as a continuous variable between zero (behaviours typically ascribed to men) to 100 (behaviours typically ascribed to women) was applied to measure the effect of gender on health outcomes [21]. Their gender score represents a summary of multiple variables comprising the four dimensions of gender and, hence, a pragmatic instrument to measure gender. The fact that the score considers gender as a bipolar, one-dimensional continuum offers a methodological advantage over more complex instruments, as it allows to include only one variable in statistical models as opposed to multiple single variables, which may lower statistical power and make the interpretation of results more difficult. The gender score is based on the short version of a questionnaire (appended in Supplementary Figure S2), which comprises a number of gender-related items including employment status, perceived social standing, housework responsibility status, education level, social support, domestic stress level as well as the Bem sex-role inventory, a measure used to assess gender roles [21-23]. Given that gender variables are time- and context-sensitive, we chose this instrument as it has been applied and validated in Switzerland in recent years [21,23]. The gender-related variables were included in a previously described logistic regression model using biological sex as the dependent variable [21]. The identified gender-related variables served as predictors to estimate the ‘probability of an individual being a woman’ which was named gender score. The mean gender score in our study population was 46.2 ± 25.9. As gender and sex usually overlap [24], the gender score and biological sex were correlated in our study (Pearson r = 0.52; 95% CI: 0.49–0.55; p < 0.001), which is consistent with data (r = 0.62) reported previously by Pelletier et al. [21]

### Statistical analyses

We defined the primary outcome measure of our analysis as the persistence of at least one COVID-19-related somatic symptom for at least 12 weeks after their first infection [1]. We performed biological sex comparisons using an independent t-test, a Mann–Whitney U test, or a chi-squared test, as appropriate. We applied a Cox proportional hazards regression with a robust variance estimator to estimate risk ratios (RR) by setting the follow-up time to a constant value to all subjects with a backward selection method to explore an association between factors and PASC [25]. A p value of less than 0.05 was used to select variables from univariate testing, and important variables such as age, sex and gender score were forced to remain in the model. Considering the multicollinearity between the gender score and the gender-sensitive sociocultural variables that were acquired to construct the gender score, we performed the multivariable analysis separately on two models. Model 1 included age, sex, gender score and other factors; Model 2 included age, sex, single features of the gender score (sociocultural variables) and other factors. After the final model was developed, we tested the interaction between sex and other variables in the model. If no interaction was presented, this term was removed from the model. We considered the Akaike information criterion (AIC) when comparing the model selection. Missing data in sociocultural and economic variables were less than 3% and can be assumed missing completely at random, therefore imputation was not performed in this study. Statistical testing was done within an exploratory framework at a two-sided significance level of α = 0.05. We performed all statistical tests using Stata IC15 (StataCorp, 2017, College Station, US).

## Results

### Patient baseline characteristics

The final study cohort comprised 2,856 individuals (1,307 (45.8%) female study participants and 1,549 (54.2%) male study participants, Table 1) and was stratified by biological sex and severity of acute illness (outpatients and hospitalised patients). The mean age (± standard deviation (SD)) of the overall study sample was 44.2 ± 16.8 years. Females were younger than males (42.7 ± 16.4 vs 45.4 ± 17.0 years) and had a lower body mass index (24.5 ± 5.2 vs 26.3 ± 4.4 kg/m^2^). The average number of cardiovascular risk factors was lower in females as compared with males. Accordingly, females less often had pre-existing cardiovascular disease than males (6.0% vs 13.7%), while females more often had mental disease (5.0% vs 3.4%) or autoimmune disorders (8.5% vs 5.0%). The average number of reported symptoms during primary infection was higher in females than males (5.3 ± 2.3 vs 4.4 ± 2.2; p < 0.001), with females reporting anosmia/dysosmia, ageusia/dysgeusia, gastrointestinal symptoms, dyspnoea or fatigue more often, while males more often presented with fever (Table 1). Notably, mean age, the frequency of comorbidities, cardiovascular risk factors and medications was higher in inpatients than outpatients (Table 1).

**Table 1:** Baseline characteristics of the study population. Stratification by biological sex and severity of acute illness (outpatients and hospitalised patients), Switzerland, February–December 2020 (n = 2,856)

	Overall							Outpatients					Inpatients				
	Total n = 2,856		Male n = 1,549		Female n = 1,307			Male n = 1,201		Female n = 1,130			Male n = 348		Female n = 177		
Distribution	Mean	SD	Mean	SD	Mean	SD	p value	Mean	SD	Mean	SD	p value	Mean	SD	Mean	SD	p value
Age (years)	44.2	16.8	45.4	17.0	42.7	16.4	<0.001	40.6	14.4	40.0	14.6	0.33	62.3	14.4	59.8	17.1	0.086
BMI (kg/m^2^)	25.5	4.8	26.3	4.4	24.5	5.2	<0.001	25.8	4.1	23.9	4.7	<0.001	28.0	4.7	28.5	6.3	0.35
Number of CVRFs	0.7	1.0	0.8	1.1	0.5	0.9	<0.001	0.5	0.9	0.4	0.7	<0.001	1.8	1.3	1.4	1.3	0.003
Frequency	n	%	n	%	n	%	p value	n	%	n	%	p value	n	%	n	%	p value
Cardiovascular risk factors																	
Hypertension	601	21.0	410	26.5	191	14.6	<0.001	185	15.4	102	9.0	<0.001	225	64.7	89	50.3	0.001
Dyslipidaemia	295	10.3	213	13.8	82	6.3	<0.001	85	7.1	34	3.0	<0.001	128	36.8	48	27.1	0.027
Diabetes mellitus	214	7.5	158	10.2	56	4.3	<0.001	58	4.8	21	1.9	<0.001	100	28.7	35	19.8	0.026
Family history of CAD	53	1.9	36	2.3	17	1.3	0.043	10	0.8	10	0.9	0.89	26	7.5	7	4.0	0.12
Present smoking	210	7.4	124	8.0	86	6.6	0.15	102	8.5	78	6.9	0.15	22	6.3	8	4.5	0.40
Obesity (BMI ≥ 30 kg/m^2^)	526	18.4	306	19.8	220	16.8	0.045	182	15.2	153	13.5	0.27	124	35.6	67	37.9	0.62
Pre-existing conditions																	
Mental disorder	119	4.2	53	3.4	66	5.0	0.030	28	2.3	30	2.7	0.62	25	7.2	36	20.3	<0.001
Autoimmune/rheumatoid disease	189	6.6	78	5.0	111	8.5	<0.001	35	2.9	75	6.6	<0.001	43	12.4	36	20.3	0.016
Chronic pulmonary disease	285	10.0	147	9.5	138	10.6	0.34	77	6.4	101	8.9	0.022	70	20.1	37	20.9	0.83
Neurological disease	142	5.0	73	4.7	69	5.3	0.49	23	1.9	41	3.6	0.011	50	14.4	28	15.8	0.66
Cancer	133	4.7	82	5.3	51	3.9	0.079	31	2.6	27	2.4	0.77	51	14.7	24	13.6	0.73
Cardiovascular disease	291	10.2	212	13.7	79	6.0	<0.001	79	6.6	41	3.6	0.001	133	38.2	38	21.5	<0.001
Medications before COVID-19																	
Cardiovascular drugs	531	18.6	361	23.3	170	13.0	<0.001	154	12.8	91	8.1	<0.001	207	59.5	79	44.6	0.001
Asthma treatment	123	4.3	62	4.0	61	4.7	0.38	33	2.7	44	3.9	0.12	29	8.3	17	9.6	0.63
Analgesics	298	10.4	124	8.0	174	13.3	<0.001	71	5.9	128	11.3	<0.001	53	15.2	46	26.0	0.003
Immunosuppressive medication	69	2.4	33	2.1	36	2.8	0.28	13	1.1	23	2.0	0.062	20	5.7	13	7.3	0.48
Antidepressants	73	2.6	28	1.8	45	3.4	0.006	21	1.7	32	2.8	0.080	7	2.0	13	7.3	0.003
Thyroid hormones	65	2.3	8	0.5	57	4.4	<0.001	7	0.6	48	4.2	<0.001	1	0.3	9	5.1	<0.001
Antiandrogens	11	0.4	11	0.7	0	0.0	0.002	8	0.7	0	0.0	0.006	3	0.9	0	0.0	0.22
Symptoms at first infection																	
Anosmia/dysosmia	1,523	53.3	729	47.1	794	60.7	<0.001	607	50.5	717	63.5	<0.001	122	35.1	77	43.5	0.059
Fever	1,515	53.0	872	56.3	643	49.2	<0.001	636	53.0	530	46.9	0.003	236	67.8	113	63.8	0.36
Gastrointestinal symptoms	556	19.5	235	15.2	321	24.6	<0.001	143	11.9	256	22.7	<0.001	92	26.4	65	36.7	0.015
Dyspnoea	709	24.8	345	22.3	364	27.9	<0.001	171	14.2	270	23.9	<0.001	174	50.0	94	53.1	0.50
Cough	1,362	47.7	745	48.1	617	47.2	0.64	558	46.5	511	45.2	0.55	187	53.7	106	59.9	0.18
Fatigue	2,076	72.7	1,055	68.1	1,021	78.1	<0.001	813	67.7	886	78.4	<0.001	242	69.5	135	76.3	0.11
Ageusia/dysgeusia	1,490	52.2	712	46.0	778	59.5	<0.001	576	48.0	692	61.2	<0.001	136	39.1	86	48.6	0.037

BMI: body mass index; CAD: coronary artery disease; CVRF: cardiovascular risk factors; SD: standard deviation.

### Socioeconomic characteristics

Gender differences in education level were most pronounced in hospitalised patients, with men in general having obtained a higher educational qualification than women (Table 2). In general, women were more often single parents than men (10.4% vs 5.8%), were more often divorced/separated (10.7% vs 7.9%) or widowed (3.4% vs 1.9%) and lived less often in a partnership than men (66.3% vs 69.1%). Men earned more often the highest income in the household (50.8% vs 19.4%), while women were more often the main person responsible for household work (39.3% vs 15.8%), had a higher responsibility for childcare/care of family members than men (score 0–6: 1.9 ± 2.3 vs 1.7 ± 2.1) and reported a higher stress level at home than men (score 0–10: 3.7 ± 2.3 vs 3.1 ± 2.0). Women more often lived alone than men (21.1% vs 18.4%). The Bem score, a measure used to assess masculine gender roles [22], was significantly higher in men than in women (5.0 ± 1.0 vs 4.9 ± 0.9). As expected, the summary gender score (0–100 with 100 being behaviours typically ascribed to women), containing all the above variables, was significantly higher in women than in men (60.8 ± 23.0 vs 33.7 ± 21.4, Table 2).

**Table 2:** Socioeconomic characteristics of the study population. Stratification by biological sex and severity of acute illness (outpatients and hospitalised patients), Switzerland, February–December 2020 (n = 2,856)

Sociocultural-and economic variables^a^	Overall							Outpatient					Inpatients				
	Total n = 2,856		Male n = 1,549		Female n = 1,307		p value	Male n = 1,201		Female n = 1,130		p value	Male n = 348		Female n = 177		p value
	n	%	n	%	n	%		n	%	n	%		n	%	n	%	
Healthcare worker																	
Yes	564	19.9	176	11.4	388	29.9	<0.001	155	12.9	358	31.9	<0.001	21	6.1	30	17.0	<0.001
No	2,277	80.1	1,367	88.6	910	70.1		1,043	87.1	764	68.1		324	93.9	146	83.0	
Education																	
No education qualification	181	6.4	97	6.3	84	6.4	0.13	60	5.0	47	4.2	0.041	37	10.8	37	21.1	<0.001
Primary education	197	6.9	91	5.9	106	8.1		58	4.9	79	7.0		33	9.6	27	15.4	
Secondary education/vocational degree	1,126	39.7	610	39.7	516	39.6		428	35.9	435	38.6		182	52.9	81	46.3	
University or technical college degree	1,335	47.0	738	48.0	597	45.8		646	54.2	567	50.3		92	26.7	30	17.1	
Marital status																	
Married/partnership	1,924	67.8	1,061	69.1	863	66.3	0.003	800	67.1	751	66.7	0.011	261	76.1	112	63.6	<0.001
Divorced/separated	260	9.2	121	7.9	139	10.7		85	7.1	116	10.3		36	10.5	23	13.1	
Single	580	20.4	324	21.1	256	19.7		295	24.7	241	21.4		29	8.5	15	8.5	
Widowed	73	2.6	29	1.9	44	3.4		12	1.0	18	1.6		17	5.0	26	14.8	
Parenthood																	
Two-parent family	1,648	58.3	957	62.5	691	53.4	<0.001	690	57.9	579	51.4	<0.001	267	78.8	112	66.3	0.006
Single-parent family	224	7.9	89	5.8	135	10.4		67	5.6	113	10.0		22	6.5	22	13.0	
No children	954	33.8	485	31.7	469	36.2		435	36.5	434	38.5		50	14.7	35	20.7	
Income																	
Earns highest income in household	1,028	36.4	775	50.8	253	19.4	<0.001	584	49.0	227	20.1	<0.001	191	57.4	26	14.9	<0.001
Earns lowest income in household	804	28.5	247	16.2	557	42.8		208	17.4	470	41.7		39	11.7	87	50.0	
Equal between partners	439	15.5	222	14.6	217	16.7		164	13.8	188	16.7		58	17.4	29	16.7	
Lives alone	555	19.6	281	18.4	274	21.1		236	19.8	242	21.5		45	13.5	32	18.4	
Main person responsible for household work																	
No	681	24.1	515	33.7	166	12.8	<0.001	372	31.2	145	12.9	<0.001	143	42.3	21	12.0	<0.001
Yes	751	26.6	241	15.8	510	39.3		196	16.5	433	38.6		45	13.3	77	44.0	
Equal distribution between partners	952	33.7	550	36.0	402	31.0		435	36.5	352	31.3		115	34.0	50	28.6	
Single household	443	15.7	223	14.6	220	16.9		188	15.8	193	17.2		35	10.4	27	15.4	
Other gender variables	Mean	SD	Mean	SD	Mean	SD	p value	Mean	SD	Mean	SD	p value	Mean	SD	Mean	SD	p value
Main responsibility for childcare/care of family members (score 0–6)	1.8	2.2	1.7	2.1	1.9	2.3	0.004	1.7	2.1	1.9	2.3	0.056	1.6	2.0	2.2	2.2	0.004
Average domestic stress level (score 0–10)	3.4	2.2	3.1	2.0	3.7	2.3	<0.001	3.1	2.0	3.7	2.3	<0.001	2.8	2.0	3.8	2.5	<0.001
Bem score [22]	4.9	1.0	5.0	1.0	4.9	0.9	<0.001	5.0	0.9	4.9	0.9	0.003	4.9	1.1	4.6	1.2	0.007
Gender score (0 = masculine, 100 = feminine) [23]	46.2	25.9	33.7	21.4	60.8	23.0	<0.001	34.7	21.2	60.2	23.1	<0.001	29.8	22.1	65.0	21.9	<0.001

Bem score: measure used to assess masculine gender roles; SD: standard deviation.

^a^ Sociocultural-and economic variables have missing data between 0.6% and 2.3%, and the missing data in the gender score was 10%.

### Acute disease characteristics in hospitalised individuals

Among hospitalised patients (n = 525 (18.4% of total study population), n = 177 (33.7%) females), routine laboratory markers of inflammation including C-reactive protein level, procalcitonin, neutrophil:lymphocyte ratio and ferritin levels were all higher in males than in females during primary infection (Table 3). Similarly, males had higher levels of indicators of organ injury such as creatinine, liver transaminases, cardiac biomarkers and lactate levels than females, and experienced more often than females respiratory, renal, thromboembolic or neurological complications (Table 3). Notably, males obtained more often anti-inflammatory or antiviral treatment such as corticosteroids (41.7% vs 32.2%; p = 0.035) or remdesivir (25.9% vs 18.1%; p = 0.046, Table 3).

**Table 3:** Acute disease characteristics in hospitalised individuals. Stratification by biological sex, Switzerland, February–December 2020 (n = 525)

	Total n = 525		Male n = 348		Female n = 177		
Clinical parameters at first day in-hospital	Mean	SD	Mean	SD	Mean	SD	p value
CURB-65^a^	1.7	1.3	1.7	1.3	1.6	1.3	0.26
MAP (mmHg)	79.6	14.7	79.8	14.7	79.3	14.8	0.73
Heart rate (beats/minute)	90.0	24.4	90.2	24.4	89.5	24.4	0.74
Respiratory rate (breaths/minute)	25.5	8.1	25.7	8.6	25.2	7.2	0.49
P/F ratio (mmHg)	277.5	115.1	269.3	114.3	294.1	115.4	0.021
Oxygen saturation (SpO_2_) (%)	90.4	5.8	90.1	5.8	91.1	5.8	0.073
Body temperature (°C)	38.0	1.0	38.0	1.0	37.8	0.9	0.005
Disease course of acute COVID-19	n	%	n	%	n	%	p value
Respiratory complications	412	78.5	285	81.9	127	71.8	0.008
Invasive ventilation	123	23.4	89	25.6	34	19.2	0.10
Haemodynamic support	126	24.0	92	26.4	34	19.2	0.067
Cardiac complications	70	13.3	50	14.4	20	11.3	0.33
Renal complications	93	17.7	70	20.1	23	13.0	0.043
Thromboembolic complications	50	9.5	41	11.8	9	5.1	0.013
Neurological complications	84	16.0	66	19.0	18	10.2	0.009
Medical treatment of acute COVID-19	n	%	n	%	n	%	p value
Corticosteroids	202	38.5	145	41.7	57	32.2	0.035
Ritonavir/lopinavir	77	14.7	54	15.5	23	13.0	0.44
Remdesivir	122	23.2	90	25.9	32	18.1	0.046
Tocilizumab	43	8.2	33	9.5	10	5.6	0.13
Chloroquine/hydroxychloroquine	122	23.2	85	24.4	37	20.9	0.37
Slow onset of acute COVID-19	244	46.5	155	44.5	89	50.3	0.21
Laboratory results at first day in-hospital	Median	IQR	Median	IQR	Median	IQR	p value
Leucocytes (G/L)	6.7	5–9	6.7	5–9	6.6	5–9	0.70
Lymphocytes (%)	15.2	9–23	13.8	8–21	17.8	11–27	<0.001
Neutrophils (%)	75.0	66–83	76.3	68–84	72.1	61–81	0.003
Ratio neutrophils:lymphocytes	5.0	2.9–9.6	5.6	3–10	4.2	2–8	0.002
CRP (mg/L)	58.0	21–123	62.0	24–126	43.2	16–115	0.020
Procalcitonin (µg/L)	0.1	0.1–0.3	0.1	0.1–0.3	0.1	0.1–0.2	<0.001
Haemoglobin (lowest value) (g/L)	133.0	119–144	135.5	124–148	127.0	116–138	<0.001
Fibrinogen (g/L)	198.5	149–266	190.0	138–252	217.0	165–276	<0.001
ALAT (U/L)	33.0	23–52	35.5	25–54	29.5	19–45	<0.001
ASAT (U/L)	40.0	28–56	42.0	29–60	36.0	26–49	0.004
Bilirubin (µmol/L)	8.0	6–11	8.7	7–12	7.2	4–10	<0.001
Creatinine (µmol/L)	82.0	67–103	88.0	75–108	68.0	57–86	<0.001
Fibrinogen (g/L)	4.9	3.8–6.1	4.8	4–6	5.2	4–7	0.33
Troponin-T (ng/L)	12.0	7.0–24.0	13.0	8–26	10.0	5–22	0.007
Creatine kinase (U/L)	86.5	52–178	110.5	61–203	61.5	44–119	<0.001
Lactate dehydrogenase (U/L)	346.0	259–472	361.0	268–505	318.0	245–454	0.017
Hormone levels^b^	n = 256		n = 157		n = 99		p value
	Mean	SD	Mean	SD	Mean	SD	
Cortisol (nmol/L)	422.9	293.9	422.1	303.7	424.2	279.3	0.95
Oestradiol (E2) (pmol/L)	126.2	146.6	107.0	80.4	157.3	211.4	0.009
Testosterone (nmol/L)	4.0	5.1	5.9	5.6	0.9	1.3	<0.001
Progesterone (ng/mL)	0.8	1.3	0.8	1.3	0.8	1.3	0.94
Ratio testosterone:oestradiol	4.8	5.7	6.8	6.2	1.1	1.2	<0.001

ALAT: alanine aminotransferase; ASAT: aspartate aminotransferase; CRP: C-reactive protein; IQR: interquartile range; MAP: mean arterial pressure; P/F ratio: PaO_2_/FiO_2_ ratio; SD: standard deviation.

^a^ CURB-65: confusion, urea nitrogen, respiratory rate, blood pressure, ≥ 65 years.

^b^ Assessed only in patients who agreed to provide samples for hormone measurement.

### Hormone levels

Among patients who agreed to provide blood samples for hormone measurements (n = 256, n = 99 (38.7%) females), cortisol or progesterone levels did not differ significantly between females and males, while the testosterone:oestradiol ratio was significantly higher in males as compared with females (p < 0.001) (Table 3).

### Post-acute sequelae of SARS-CoV-2 infection: prevalence and type of symptoms

**Figure 1:**
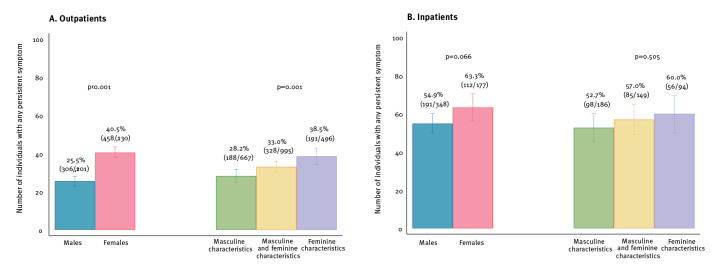
Percentage of patients reporting any persisting symptom following acute COVID-19 disease, either mild (A) or moderate/severe (B), Switzerland, June 2020–June 2021 (n = 2,856)

During a mean follow-up time of 203 ± 76 days (males: 200 ± 76 days vs females: 206 ± 77 days) 1,067 (37.4%) individuals reported at least one somatic symptom that persisted beyond 12 weeks following primary infection. The prevalence of PASC was higher in hospitalised patients than in outpatients (57.7% vs 32.8%; p < 0.001). Among outpatients, females reported more often than males at least one persistent somatic symptom (40.5% vs 25.5%; p < 0.001). Similarly, the prevalence of PASC was higher in outpatients scoring within the highest tertile of the gender score (= feminine characteristics) as compared with lower tertiles (p = 0.001) (Figure 1A). However, these significant sex and gender differences were no longer evident in patients who had been hospitalised during primary infection (Figure 1B).

Data are stratified by sex as a binary variable (left) or tertiles of gender (right). Data are presented as incidence rates and 95% confidence interval.

**Figure 2:**
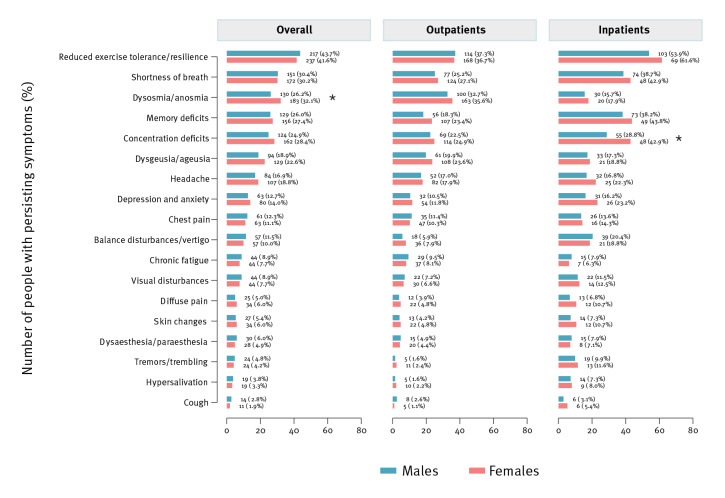
Persistent symptoms reported at follow-up stratified by sex and symptom, Switzerland, June 2020–June 2021 (n = 2,856)

The most frequently reported PASC symptom was reduced exercise tolerance and resilience in both sexes, which was reported by 43.7% of males and 41.6% of females (p = 0.49), followed by shortness of breath (30.4% of males and 30.2% of females; p = 0.94) and dysosmia/anosmia (26.2% of males and 32.1% of females; p = 0.033, Figure 2). No significant sex difference in the type of PASC symptoms, except for dysosmia/anosmia, was seen in the overall cohort and outpatients. Concentration deficits were more often reported by female inpatients as compared with male inpatients (42.9% vs 28.8%; p = 0.013, Figure 2).

Data are presented as percentage of patients reporting persistent symptoms.

### Predictors of post-acute sequelae of SARS-CoV-2 infection: sex vs gender

**Figure 3:**
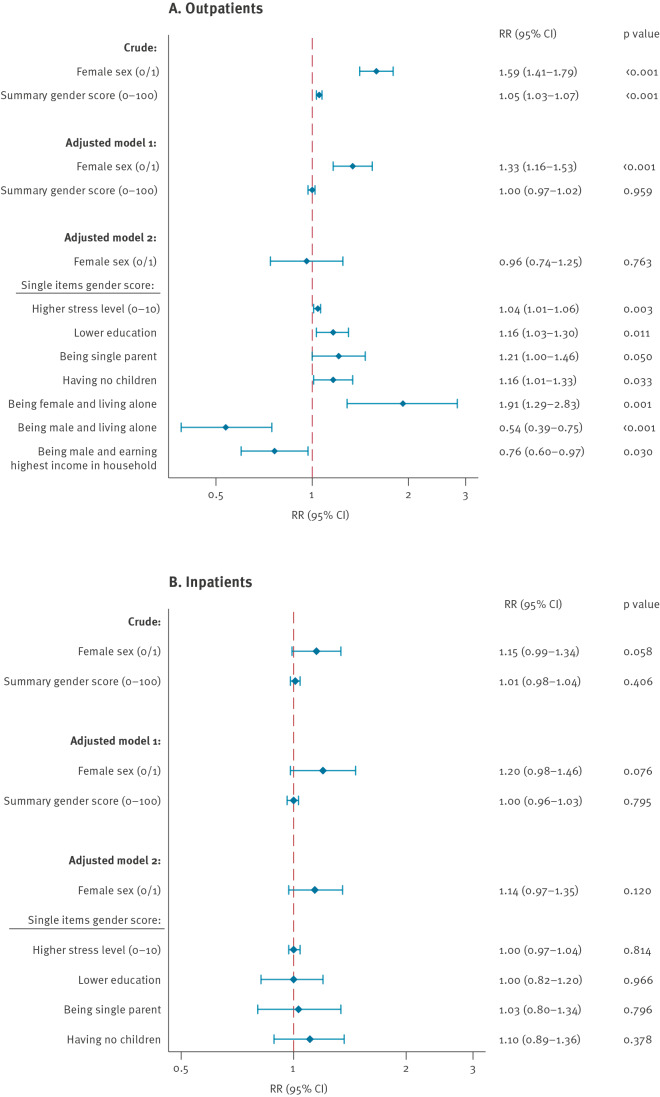
Risk and protective factors associated with any persistent symptom following SARS-CoV-2 infection in outpatients (A) and hospitalised patients (B), Switzerland, February 2020–June 2021 (n = 2,856)

CI: confidence interval; RR: risk ratio.

In a crude analysis, both female sex (RR = 1.59; 95% CI: 1.41–1.79; p < 0.001) and the gender score (RR = 1.05; 95% CI: 1.03–1.07; p < 0.001) were significantly associated with PASC in outpatients (Figure 3A). In outpatients, following multivariable adjustment, biological female sex (RR = 1.33; 95% CI: 1.16–1.53; p < 0.001), but not the gender score, remained a significant predictor of PASC (Model 1, Figure 3A). However, when the single features, instead of the summary gender score, were introduced into the model, biological sex (RR = 0.96; 95% CI: 0.74–1.25; p = 0.763) was outperformed by gender-related factors such as a higher stress level (RR = 1.04; 95% CI: 1.01–1.06; p = 0.003), lower education (RR = 1.16; 95% CI: 1.03–1.30; p = 0.011), being female and living alone (RR = 1.91; 95% CI: 1.29–2.83; p = 0.001) or having no children (RR = 1.16; 95% CI: 1.01–1.33; p = 0.033, Model 2, Figure 3A). In addition, we observed a trend towards a higher risk for PASC for being single parent (RR = 1.21; 95% CI: 1.00–1.46; p = 0.050). Conversely, being male and living alone (RR = 0.54; 95% CI: 0.39–0.75; p < 0.001) or earning the highest income in the household (RR = 0.76; 95% CI:0.60–0.97; p = 0.030) were independently associated with a lower risk of PASC (Figure 3A). 

Forest plots derived from Cox proportional hazards regression models depicting risk ratios and 95% confidence intervals. For the gender score the risk per unit changes by 10 points.

In hospitalised patients, neither sex nor gender were associated with the occurrence of PASC (Figure 3B). The full models for both outpatients and inpatients are provided in Supplementary Tables S3 and S4. Notably, despite being independently associated with the occurrence of PASC, the RRs presented in the outpatient model were relatively small, except for the interaction variable ‘being female and living alone’ (RR = 1.91). In addition, neither age nor sex hormone levels or their ratios were significant predictors of PASC in our models (Supplementary Table S4). Finally, there was no association between hormone intake/replacement (hormonal contraception in 278 women, postmenopausal hormone replacement in 35 women, fertility treatments in nine women, regular testosterone intake in 22 men, daily intake of phytoestrogens in 69 women and 33 men) or hormone deprivation therapies (anti-oestrogen therapy for breast/gynaecological cancer in 17 women, anti-androgenic treatment for prostate cancer in five men) and PASC in our study (data not shown).

## Discussion

Our study reports that, unlike biological sex, sociocultural parameters, that differ in prevalence between women and men, were risk predictors of PASC and may explain, at least in part, the female propensity towards a higher risk of PASC, despite their lower risk of severe acute illness. Independent risk predictors of PASC in both sexes comprise a higher stress level, lower education, being a single parent or having no children. We identified ‘living alone’ as a PASC predictor unique to women, while earning the highest income in the household was a protective factor unique to men. We also demonstrate that, once patients were hospitalised during SARS-CoV-2 infection, sex differences in the incidence of PASC were no longer evident.

Our study adds to increasing evidence indicating that COVID-19 sex disparities cannot solely be explained by sex-specific biological mechanisms and are also explained by gendered patterns in contextual factors [26]. In line with this notion, age-related changes in hormone status, hormone replacement or hormone deprivation therapies were not associated with PASC in either sex in our study, although it has recently been proposed that the symptoms of PASC may overlap with those of perimenopause [27]. Similarly, psychosocial and behavioural factors and their interaction with sex (e.g. living alone and being female) remained among the strongest predictors of PASC, even when a large amount of biological variables derived from our well-characterised study cohort were included in the model. Consistent with our conclusion, increasing evidence suggests that substantial variation in the magnitude and direction of COVID-19 sex disparities exists across geographical localities, among racial and ethnic groups and over time, all of which indicate that the analysis of gendered contextual factors might offer important insights into outcomes and should be considered alongside sex differences as COVID-19 research moves forward [26,28,29].

In our study population the reported domestic stress level was significantly higher in women than in men. This observation is consistent with previous reports indicating that the burden of psychosocial stress has increased more in women than in men during the pandemic. Indeed, women have been disproportionately affected by imposed quarantine and lockdown measures given that typical feminine roles such as parenting, home-schooling and other caring duties are still predominantly assumed by women [30]. Isolation at home measures along with financial and security concerns can put an additional strain on women, who, more often than men, led single parent families, lived alone or had a lower education level in our study. Although lower education or increased domestic stress were significant predictors of PASC in both men and women in our study, the higher prevalence of these risk factors in the female study population might mirror not only the vulnerable socioeconomic positions of women during the pandemic but also highlights that their ability to return to work might be further impeded by the chronicity of symptoms of PASC. However, while the link between these variables and the occurrence of PASC provides important information, it is notable that sociocultural gender consists of intertwined dimensions [31]. Hence, it has to be taken into account that single variables of the gender score cannot reflect the multiple dimensions provided by the gender score and might be seen as simple sociodemographic variables.

The reasons for the differential impact of sex and gender in outpatients vs hospitalised patients can only be hypothesised, but might be attributed to the substantial differences in baseline characteristics between hospitalised patients and outpatients. In fact, compared with outpatients, hospitalised patients were significantly older (61.5 ± 15.4 years vs 40.3 ± 14.5 years in outpatients) and had more frequently comorbidities and cardiovascular risk factors. As gender roles and attributes largely depend on age and generation, the age difference between in- and outpatients might have impacted the association between gender-related factors and study outcomes. The high prevalence of comorbidities was seen in both female and male hospitalised patients resulting in a more homogenous study population as compared with outpatients, where sex differences were more obvious. Accordingly, variables such as invasive ventilation during acute illness, known hypertension, or specific symptoms at presentation for acute COVID-19 were all better predictors for PASC in hospitalised patients than sex or gender and might drive their risk for long-term consequences or the disease. Our differential results in hospitalised vs outpatients also support the hypothesis that PASC in hospitalised individuals might have different aetiologies than PASC in outpatients and may resemble post-hospital-syndrome or post-intensive-care-syndrome (PICS). Nevertheless, it is notable that the vast majority of PASC patients were younger (mean age: 47 years) and were only mildly ill during their first infection, thereby imposing a substantial burden on healthcare systems and economies.

The strength of our analysis consists of its near-complete, geographically defined cohort with availability of more than 200 clinical, laboratory, socioeconomic and psychosocial variables, the capture of a wide spectrum of post-COVID-19 symptoms, the availability of SARS-CoV-2 swab test results in all study participants and the multicentre design permitting to include both outpatients with mild disease as well as hospitalised patients. Data characterising primary infection were collected during ambulatory visits or hospitalisation, thereby minimising recall bias. However, our study also has several limitations related to its cross-sectional and observational design. Firstly, although the variables in our study covered many aspects of sex- and gender-specific demographic, behavioural and contextual characteristics, residual confounding due to unmeasured parameters in our dataset is possible. Secondly, self-selection or other biases may have occurred if individuals who are more concerned with their health were more likely to participate. Thirdly, our study was conducted in Switzerland, a high-income country with a high gender equality index [32]. Given that gender-related characteristics are culturally sensitive, our observations may not be extrapolated to other societies and geographical regions. Similarly, the Swiss population is ca 95% white, with only very small minority groups. Data derived from this ethnically and racially homogenous population allows for a focused consideration of gender in COVID-19 outcomes, however, at the expense of more complex interactions of other factors with disease outcomes. Fourthly, recent studies indicate that vaccination might protect from PASC [4]. As vaccination started after recruitment was completed, we were unable to address this issue. Finally, our study does not allow to assess the impact of SARS-CoV-2 variants on the epidemiology and severity of PASC as this information was not collected.

## Conclusion

Taken together, while we did not observe major sex and gender differences in PASC symptom presentation, the incidence of PASC was substantially higher in women than in men in our Swiss cohort of almost 3,000 patients. Although biological variables may have a role in explaining sex disparities in acute COVID-19 illness, our study suggests an impact of societally constructed characteristics, and their interaction with biological sex, in producing sex and gender differences in PASC. Currently, a symptom-specific approach to treat PASC is recommended, however, our data imply that a tailored gender-sensitive approach of healthcare services may be required to support the needs of individuals affected by PASC. Indeed, many predictor variables of PASC identified in the present study are targets of interventions aiming at stress coping and social support. Also, the reported PASC risk factors can be easily identified at an early stage of disease by taking a thorough patient history without additional blood sampling or extensive diagnostic testing. Further research will be needed to determine if interventions targeted at these factors could improve outcomes. Finally, the fact that single features of the gender score, but not the summary gender score itself, were major predictors of PASC was surprising as the gender score was designed to provide a more holistic view of the impact of gender than single variables. Potential explanations for our finding comprise the fact that our study population was younger than the standard populations in which the gender score was validated, as well as the impact of differential environmental and health-related factors operating during a major pandemic on our study endpoints. Hence, our study emphasises the need to further advance instruments for the operationalisation of gender which may require adaptation to specific societies, age groups and disease conditions. Accordingly, we will use the data collected during this study to further optimise the gender score for contemporary research questions in future projects in Switzerland.

## Supplementary Data

1022000 Supplement
