# Supplementary material for: Impact of sex and gender on post-COVID-19 syndrome, Switzerland, 2020
Source: Euro Surveill. 2024 Jan 11;29(2):2300200. doi: 10.2807/1560-7917.ES.2024.29.2.2300200 (PMC10785203; doi:10.2807/1560-7917.ES.2024.29.2.2300200)
Supplement: Supplement [file 23-00200_GEBHARD_Supplement.pdf]

Supplementary Materials

This supplementary material is hosted by Eurosurveillance as supporting information alongside the article “Impact of sex and gender on post-COVID-19 syndrome, Switzerland, 2020”, on behalf of the authors, who remain responsible for the accuracy and appropriateness of the content. The same standards for ethics, copyright, attributions and permissions as for the article apply. Supplements are not edited by Eurosurveillance and the journal is not responsible for the maintenance of any links or email addresses provided therein.

S 1: Flowchart depicting patient selection. ICU, intensive care unit; IMC, intermediate care unit.

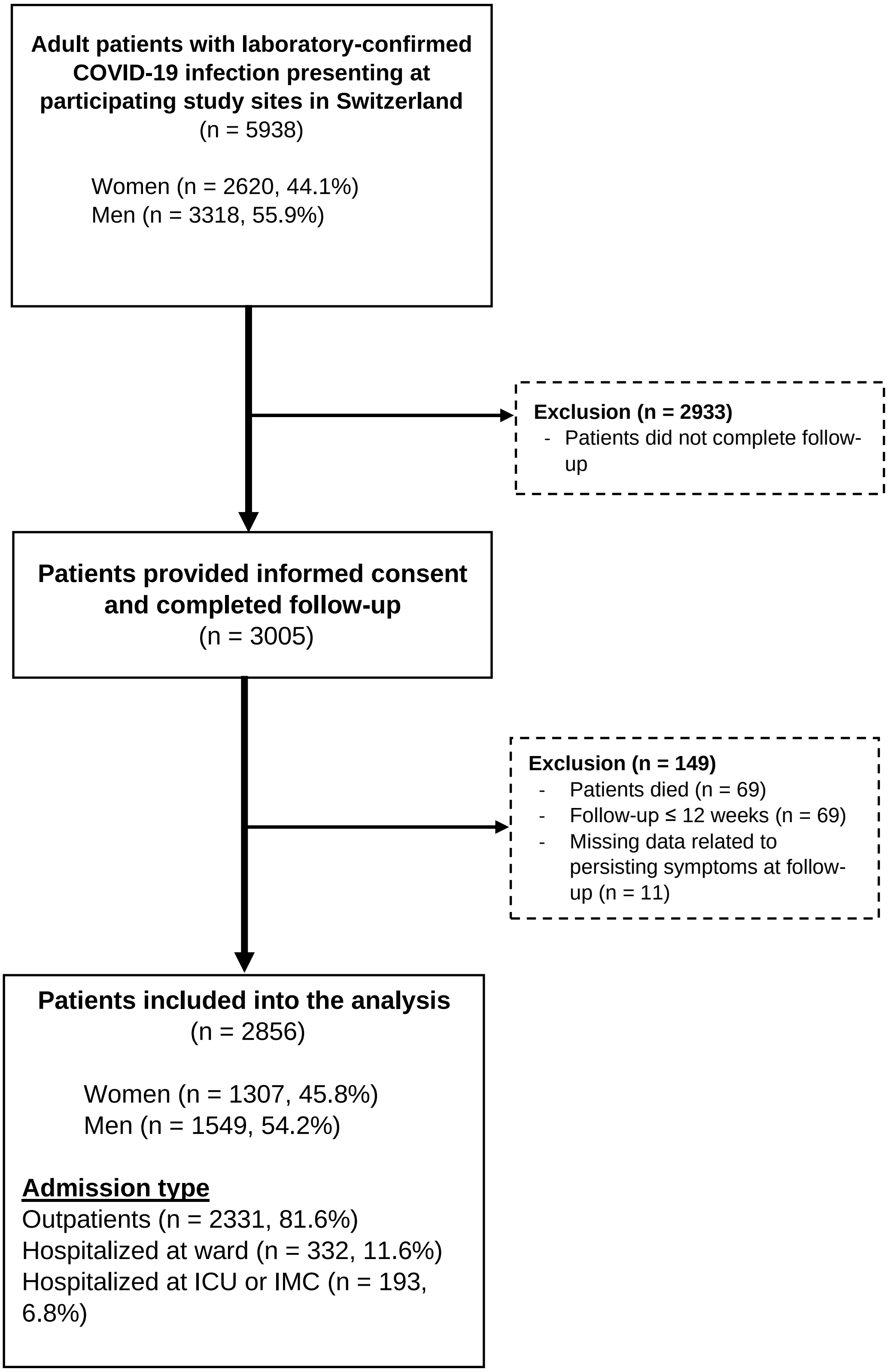

S 2: Gender-related questions included in the questionnaire.

Are/were you a single parent?	◯ Yes<br>◯ No<br>◯ I do not have children
What is your marital status?<br>(Select **only one** answer)	◯ Married, living together with spouse<br>◯ Married, but permanently living apart<br>◯ Registered same-sex partnership, living together<br>◯ Registered same-sex partnership, living apart<br>◯ Single, permanent partnership<br>◯ Single, living alone<br>◯ Divorced / partnership dissolved<br>◯ Widowed / life partner deceased
What educational qualifications have you gained? Only tick the highest degree that you gained.<br>(Select **only one** answer)	◯ No degree<br>◯ Completion of secondary education<br>◯ Apprenticeship / vocational qualification, specialist baccalaureate, Higher school certificate<br>◯ College, technical college or university
Do you earn the highest income in your household?	◯ Yes<br>◯ No<br>◯ The partners contribute in roughly equal shares<br>◯ I live alone
Are you the main person responsible for the household work in your household?	◯ Yes<br>◯ No<br>◯ The partners contribute in roughly equal shares<br>◯ I live alone

On a scale of 1 to 10, how stressed are you at home?

Assign an appropriate number to yourself:

(1) = No stress, (10) = Maximum stress

	(1)	(2)	(3)	(4)	(5)	(6)	(7)	(8)	(9)	(10)
Stress at home	◯	◯	◯	◯	◯	◯	◯	◯	◯	◯

To what extent are you responsible for the care or upbringing of children or grandchildren and/or the care of others living in your household?

Assign yourself an appropriate number:

(0) = Not applicable

(1) = I have no responsibility

(6) = I take full responsibility

	(0)	(1)	(2)	(3)	(4)	(5)	(6)
Responsibility	◯	◯	◯	◯	◯	◯	◯

(1) = Never true or almost never

(7) = True (nearly) always

I am someone who...

	(1)	(2)	(3)	(4)	(5)	(6)	(7)
... defends his own opinion.	◯	◯	◯	◯	◯	◯	◯
... has leadership qualities.	◯	◯	◯	◯	◯	◯	◯
... is independent.	◯	◯	◯	◯	◯	◯	◯
... is willing to take risks.	◯	◯	◯	◯	◯	◯	◯
... is positive.	◯	◯	◯	◯	◯	◯	◯
... is assertive.	◯	◯	◯	◯	◯	◯	◯
... has a strong personality.	◯	◯	◯	◯	◯	◯	◯
... is ready to take a stand.	◯	◯	◯	◯	◯	◯	◯
... is energetic.	◯	◯	◯	◯	◯	◯	◯
... is aggressive.	◯	◯	◯	◯	◯	◯	◯

If you assess yourself: Do you have only male or only female characteristics or both?

Assign a corresponding number to yourself:

(1) = more male characteristics

(7) = more female characteristics

	(1)	(2)	(3)	(4)	(5)	(6)	(7)
I feel that I am...	◯	◯	◯	◯	◯	◯	◯

S 3: Full prediction model for the occurrence of PASC in outpatients. Risk ratios (RR) and 95% confidence intervals (CI) are depicted. The following independent variables were included in the analysis: biological sex, gender score (Model 1) or single features of the gender score (Model 2), age, acute COVID-19 symptoms, pre-existing comorbidities, prior medications, and cardiovascular risk factors. NS = not significant.

	**Bivariate analysis**		**Model 1 (n=2158)**		**Model 2 (n=2298)**	
**Factors**	**Crude RR (95%CI)**	**p-value**	**Adjusted RR (95%CI)**	**p-value**	**Adjusted RR (95%CI)**	**p-value**
Age (years)	1.003 (1.00, 1.01)	0.212	1.00 (0.99, 1.004)	0.896	1.00 (0.99, 1.01)	0.464
Female sex	1.59 (1.41, 1.79)	<0.001	1.33 (1.16, 1.53)	<0.001	0.96 (0.74, 1.25)	0.763
Gender score (0=Masculine; 100=Feminine) x 10 scores	1.05 (1.03, 1.07)	<0.001	1.00 (0.97, 1.02)	0.959		
**CV risk factors**						
Obesity (BMI≥30 kg/m^2)	1.80 (1.42, 2.28)	<0.001	1.34 (1.16, 1.54)	<0.001	1.25 (1.08, 1.44)	0.002
**Pre-existing comorbidities**						
Mental disorder	1.77 (1.40, 2.23)	<0.001	1.35 (1.03, 1.76)	0.027	1.28 (0.99, 1.64)	0.056
Autoimmune/Rheumatoid disease	1.78 (1.50, 2.13)	<0.001	1.35 (1.12, 1.64)	0.002	1.36 (1.14, 1.63)	0.001
**Medications before COVID-19**						
Cardiovascular drugs	1.21 (1.02, 1.43)	0.028		NS		NS
Asthma treatment	1.62 (1.29, 2.02)	<0.001		NS		NS
Analgetics	1.47 (1.25, 1.73)	<0.001		NS		NS
Immunosuppressive medication	1.71 (1.27, 2.31)	<0.001		NS		NS
Antidepressants	1.64 (1.26, 2.12)	<0.001		NS		NS
Thyroid hormones	1.75 (1.38, 2.23)	<0.001		NS		NS
**Symptoms at presentation for acute COVID-19**						
Anosmia/dysosmia	1.94 (1.70, 2.22)	<0.001	1.74 (1.52, 2.00)	<0.001	1.69 (1.48, 1.93)	<0.001
Gastrointestinal symptoms	1.76 (1.57, 1.99)	<0.001	1.32 (1.16, 1.50)	<0.001	1.33 (1.18, 1.50)	<0.001
Dyspnea	2.13 (1.91, 2.38)	<0.001	1.58 (1.41, 1.78)	<0.001	1.60 (1.43, 1.80)	<0.001
Cough	1.35 (1.20, 1.51)	<0.001	1.12 (0.99, 1.26)	0.062	1.12 (1.00, 1.26)	0.055
Fatigue	1.83 (1.55, 2.16)	<0.001	1.44 (1.22, 1.70)	<0.001	1.44 (1.23, 1.70)	<0.001
**Sociocultural-and economic variables**						
Parenthood						
Two-parent family	1.00 (Reference)				1.00 (Reference)	
Single-parent family	1.33 (1.10, 1.61)	0.003			1.21 (1.00, 1.46)	0.050
No children	1.06 (0.94, 1.20)	0.336			1.16 (1.01, 1.33)	0.033
Education						
University or technical college degree	1.00 (Reference)				1.00 (Reference)	
Non-University or technical college degree	1.23 (1.09, 1.39)	0.001			1.16 (1.03, 1.30)	0.011
Average domestic stress level	1.08 (1.05, 1.11)	<0.001			1.04 (1.01, 1.06)	0.003
**Interaction terms**						
Female:Lives alone					1.91 (1.29, 2.83)	0.001
Male:Earns highest income in household					0.76 (0.60, 0.97)	0.030
Male:Lives alone					0.54 (0.39, 0.75)	<0.001

S 4: Full prediction model for the occurrence of PASC in hospitalized patients. Risk ratios (RR) and 95% confidence intervals (CI) are depicted. The following independent variables were included in the analysis: biological sex, gender score (Model 1) or single features of the gender score (Model 2), age, BMI, acute COVID-19 symptoms, pre-existing comorbidities, prior medications, cardiovascular risk factors, routine laboratory parameters during acute illness, hormone levels (testosterone, oestradiol, progesterone, and cortisol), and clinical data during acute illness. P/F ratio, PaO2/FiO2 ratio. NS = not significant.

	**Bivariate analysis**		**Model 1 (n=429)**		**Model 2 (n=490)**	
**Factors**	**Crude RR (95%CI)**	**p-value**	**Adjusted RR (95%CI)**	**p-value**	**Adjusted RR (95%CI)**	**p-value**
Age (years)	1.004 (1.00, 1.01)	0.147	1.003 (0.99, 1.01)	0.393	1.01 (1.00, 1.03)	0.121
Female sex	1.15 (0.99, 1.34)	0.058	1.20 (0.98, 1.46)	0.076	1.14 (0.97, 1.35)	0.120
Gender score (0=Masculine - 100=Feminine) x 10 scores	1.01 (0.98, 1.04)	0.406	1.00 (0.96, 1.03)	0.795		
**CV risk factors**						
Hypertension	1.23 (1.05, 1.44)	0.010	1.24 (1.03, 1.50)	0.023	1.19 (1.00, 1.41)	0.050
**Medications before acute COVID-19**						
Cardiovascular drugs	1.19 (1.02, 1.38)	0.024		NS		NS
Asthma treatment	1.32 (1.09, 1.59)	0.004		NS		NS
Analgetics	1.20 (1.02, 1.41)	0.030		NS		NS
**Symptoms at presentation for acute COVID-19**						
Anosmia/dysosmia	1.21 (1.05, 1.40)	0.008	1.19 (1.01, 1.40)	0.037	1.20 (1.03, 1.40)	0.021
Gastrointestinal symptoms	1.32 (1.14, 1.52)	<0.001	1.29 (1.10, 1.52)	0.001	1.27 (1.09, 1.47)	0.002
Dyspnea	1.44 (1.24, 1.68)	<0.001	1.26 (1.05, 1.52)	0.014	1.26 (1.06, 1.48)	0.007
Fatigue	1.50 (1.22, 1.83)	<0.001	1.34 (1.06, 1.69)	0.013	1.35 (1.09, 1.67)	0.006
**Medical treatment of acute COVID-19**						
Corticosteroids	1.23 (1.07, 1.43)	0.004		NS		NS
Tocilizumab	1.42 (1.19, 1.68)	<0.001		NS		NS
**Disease course of acute COVID-19**						
Invasive ventilation	1.56 (1.37, 1.78)	<0.001	1.55 (1.32, 1.82)	<0.001	1.53 (1.32, 1.77)	<0.001
**Sociocultural-and economic variables**						
Parenthood						
Two-parent family	1.00 (Reference)					
Single-parent family	1.14 (0.89, 1.45)	0.294			1.03 (0.80, 1.34)	0.796
No children	1.11 (0.92, 1.35)	0.258			1.10 (0.89, 1.36)	0.378
Education						
University or technical college degree	1.00 (Reference)					
Non-University or technical college degree	1.10 (0.91, 1.33)	0.307			1.00 (0.82, 1.20)	0.966
Average domestic stress level	1.03 (1.00, 1.06)	0.048			1.00 (0.97, 1.04)	0.814
**Laboratory and clinical parameters at 1st day in-hospital**						
Respiratory rate day 1 (per minute)	1.015 (1.007, 1.022)	<0.001		NS		NS
P/F ratio day 1	0.998 (0.997, 0.999)	<0.001		NS		NS
**Late presentation for acute COVID-19 (yes/no)**	1.16 (1.002, 1.34)	0.047	1.25 (1.06, 1.47)	0.008		NS
**Hormones (Cortisol, Estradiol, Testosterone)**						
Estradiol (E2, x 100 pmol/L)	1.08 (1.04, 1.12)	<0.001		NS		NS
**Interaction terms**						
Female:Late presentation for acute COVID-19				NS		
